# Improving Good Practices for Patient Safety in an Emergency Department Based on Multidisciplinary Training Using Simulation Techniques

**DOI:** 10.3390/nursrep15100351

**Published:** 2025-09-26

**Authors:** Francisco Javier Redondo Calvo, Victor Baladrón González, María Ángeles Tebar Betegón, Alejandro Martínez Arce, Gema Verdugo Moreno, Juan Fernando Padin, Laura Muñoz de Morales-Romero, Alberto Bermejo-Cantarero, Natalia Bejarano Ramírez

**Affiliations:** 1 Department of Anesthesiology, University General Hospital, 13005 Ciudad Real, Spain; vbaladron@sescam.jccm.es; 2 Faculty of Medicine, Universidad de Castilla-La Mancha, 13071 Ciudad Real, Spain; fernando.padin@uclm.es (J.F.P.); nbejarano@sescam.jccm.es (N.B.R.); 3 Translational Research Group, GAI of Ciudad Real, Spain, Research Institute of Castilla-La Mancha (IDISCAM), 13004 Ciudad Real, Spain; gverdugo@sescam.jccm.es; 4 Advanced Simulation Group, GAI of Ciudad Real, Spain, Research Institute of Castilla-La Mancha (IDISCAM), 13004 Ciudad Real, Spain; alexmartinezarce82@gmail.com; 5 Department of Nursing, University General Hospital, 13005 Ciudad Real, Spain; tebarbetegon@gmail.com (M.Á.T.B.); lmunozd@sescam.jccm.es (L.M.d.M.-R.); alberto.bermejo@uclm.es (A.B.-C.); 6 Department of Quality, University General Hospital, 13005 Ciudad Real, Spain; 7 Department of Emergency, University General Hospital, 13005 Ciudad Real, Spain; 8 Faculty of Nursing, Universidad de Castilla-La Mancha, 13071 Ciudad Real, Spain; 9 Department of Paediatrics, University General Hospital, 13005 Ciudad Real, Spain

**Keywords:** simulation, crisis resource management, team training, emergency nursing, debriefing, patient safety, quality improvement

## Abstract

**Background**: We present a multidisciplinary training experience based on simulation techniques and critical resource management implemented in the emergency department. **Methods**: Simulation courses/workshops were conducted with a multidisciplinary team from the Hospital Emergency Department. The timeline for their development includes a preliminary analysis of needs, objectives, and scenario design, development of the simulation course, and finally, areas of implementation. In this last phase, the teaching team prepares a document and/or report/summary of the activity in which, among other things, the aspects with the greatest capacity for improvement or the areas for implementation of safety measures are determined. A total of 112 healthcare professionals (doctors, nurses, and care assistants) participated in this training program. Its design consisted of the following stages: a preliminary analysis of training needs, the establishment of objectives and scenario design, the development of the simulation workshop, and finally, a report on areas for improvement in patient safety identified during the workshop learning process. **Results**: The workshops enabled us to identify areas for improvement and develop local protocols/recommendations aimed at improving patient safety in the emergency department, such as standardizing a protocol to guide us in managing resources in crisis situations, a protocol for airway management, a protocol for massive transfusion, and a review of the triage process. In addition, we added value by incorporating cognitive aids and visual tools into the standardization of processes. **Conclusions**: For resource management in this type of crisis in the hospital emergency setting, it is essential to use a debriefing process guided by experienced instructors after a specific experiential learning experience through simulation scenarios. This helps to contextualize and analyze the advantages and disadvantages of general recommendations.

## 1. Introduction

In complex work environments such as emergency medicine, decisions are often made under conditions of uncertainty and time pressure. Studies have shown that up to 70% of errors in these settings can be attributed to human factors [1,2,3]. One effective strategy to mitigate such errors is the application of Crisis Resource Management (CRM) principles [4].

Originally developed in aviation, CRM has demonstrated its value across various disciplines. Gaba et al. adapted the concept to healthcare, specifically in anesthesiology, under the term Crisis Management Resources in Anaesthesia [5]. CRM focuses on the coordination, utilization, and optimization of all available resources to enhance patient safety. These resources include the healthcare team, their skills and attitudes, equipment, and environmental factors. CRM begins before a crisis occurs, emphasizing that standards designed to manage acute events are equally effective in preventing them. The approach aims to detect errors early and minimize their impact. Although initially developed for perioperative care, CRM principles are highly applicable to emergency medicine [6].

Emergency settings present unique challenges compared to other specialties, including the urgency of decision making for critically ill patients, diverse and sometimes hostile intervention contexts, and the need for coordination across multiple levels of care [7,8,9].

While CRM principles may appear intuitive (see Table 1), their effective application during crises is often difficult. Clinical simulation has emerged as a powerful tool to enhance CRM competencies, enabling healthcare professionals to reflect on and integrate these principles into daily practice. The SEMES Clinical Simulation Group (E-CRM) has been instrumental in adapting CRM methodologies to emergency care, developing tools to standardize workflows and improve patient safety [7,8,9].

Simulation-based training is increasingly integrated into the continuous education of healthcare professionals—including physicians, nurses, and care assistants—in response to the growing complexity of clinical care and the need for multidisciplinary collaboration [10,11]. This methodology provides a realistic and safe environment for acquiring both technical and non-technical skills, such as leadership, coordination, and communication. These competencies are developed during the experiential phase and reinforced through structured debriefing sessions [12,13].

In this context, we advocate for a robust simulation methodology capable of generating realistic clinical scenarios that evoke emotional engagement, facilitate learning, and serve as a foundation for expert-led analysis and discussion. We hypothesize that the implementation of high-fidelity simulation training based on CRM principles in emergency departments improves team coordination and contributes to the development of standardized clinical protocols, ultimately enhancing patient safety. This paper presents the experience of the Advanced Simulation Centre within the Integrated Healthcare Management of Ciudad Real (Healthcare Service of Castilla-La Mancha, Spain), focusing on its application in the hospital emergency department.

In recent years, the increasing complexity of emergency care and the growing awareness of patient safety have underscored the need for innovative educational approaches. Traditional training methods often fall short in preparing healthcare professionals for the dynamic and unpredictable nature of emergency scenarios. In this context, simulation-based education has emerged not only as a pedagogical tool but also as a strategic resource for fostering clinical excellence and resilience. By replicating high-risk situations in a controlled environment, simulation enables teams to develop critical thinking, reinforce collaborative behaviors, and identify latent safety threats before they manifest in real clinical settings.

Therefore, the objective of this study is to describe and analyze the implementation of a structured simulation-based training program in the emergency department of a tertiary hospital, aimed at enhancing CRM competencies among multidisciplinary healthcare teams. By evaluating the design, execution, and perceived impact of these workshops, we seek to contribute to the development of effective educational strategies that improve patient safety and team performance in high-pressure clinical environments

## 2. Materials and Methods

Over a four-year period, eight simulation-based workshops were conducted with a multidisciplinary team from the hospital’s emergency department. Two workshops were held each year, in February and November. A prospective cohort of 112 healthcare professionals voluntarily participated in this training program, which was developed at the Advanced Simulation Center of the General University Hospital of Ciudad Real. The cohort included 39 physicians, 51 nurses, and 22 nursing assistants. This represents approximately 60% of the total staff working in the emergency department. Each workshop was conducted with 14 professionals, which matches the maximum capacity of the Simulation Center (12–14 participants per session). Recruitment was voluntary among staff members currently working in the emergency department, and selection was based on the order of registration on the hospital’s training platform. Previous participants were excluded from subsequent workshops. Only healthcare professionals currently working in the emergency department were eligible for inclusion in the study; prior participation in any of the workshops was considered an exclusion criterion.

The workshops were conducted in a simulation-based training environment, involving healthcare professionals and following the same work schedule in all sessions (see Appendix C). No biomedical interventions were performed, and no sensitive personal data were collected. The only evaluation conducted was a satisfaction survey completed by the participants. All individuals provided informed consent and signed confidentiality and image release agreements prior to participation. The teaching team consisted of two simulation instructors, one assistant/confederate, and one emergency department professional.

The development of these workshops followed a structured four-phase process, outlined chronologically below:

Phase 1: Preliminary Needs Analysis

A teaching team was assembled, consisting of two simulation instructors, one assistant/confederate, and two emergency department professionals. This team conducted a comprehensive analysis of training needs, considering both individual clinical expectations and team-wide requirements. Key patient safety aspects were also identified for inclusion in the training.

Phase 2: Definition of Objectives and Scenario Design

**Figure 1:**
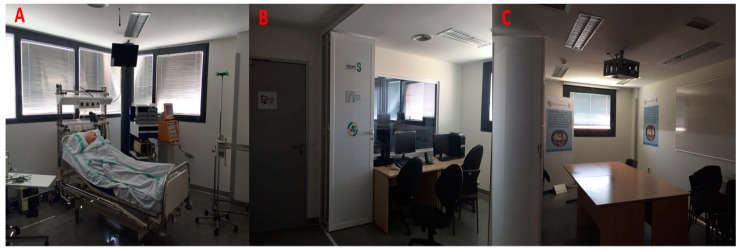
(**A**) Room where clinical scenarios are developed. (**B**) Control Room. (**C**) Debate room.

Each workshop included three high-fidelity simulation scenarios (see Table 2), with each scenario targeting two clinical skill objectives and two behavioral or CRM (Crisis Resource Management) objectives. The simulations were conducted in a fully equipped simulation suite, including a realistic clinical environment, a control room, and a debriefing room (Figure 1). A robust audiovisual system enabled real-time viewing and playback during debriefing sessions.

Phase 3: Workshop Implementation

Each training session lasted six hours and included 14 participants (4 physicians, 6 nurses, and 4 care assistants). In each workshop, three clinical scenarios were developed: a patient with sepsis, a polytrauma patient, and a patient with a difficult airway (see Appendix B). Following each scenario, a structured debriefing was conducted with the entire team. The educational methodology integrated both technical skills (e.g., manual ventilation techniques) and non-technical competencies such as leadership, communication, and teamwork, all within a multidisciplinary framework. A key innovation was the identification of systemic factors contributing to patient risk, offering a broader perspective on safety improvement.

Phase 4: Identification of Implementation Areas

After each workshop, the teaching team prepared a summary report outlining areas for improvement and potential safety interventions. These reports were submitted to the Head of Service, Unit Supervisor, and, where appropriate, the Health Risk Commission and hospital management.

At the end of the workshops, all participants were asked to complete an evaluation survey (see Table 3) to assess the course objectives, methodology, and supporting materials. Each item is rated on a scale from 0 to 5, and the results are presented as mean values and standard deviations.

## 3. Results

The development of the workshops enabled the identification opportunities and develop local protocols/recommendations aimed at improving patient safety. One of the main outcomes was the creation of a protocol for the distribution of roles and responsibilities among healthcare professionals during resuscitation scenarios. Emphasis was placed on pre-established role assignments at the onset of resuscitations, which are now updated with each shift change to ensure consistency and preparedness.

**Figure 2:**
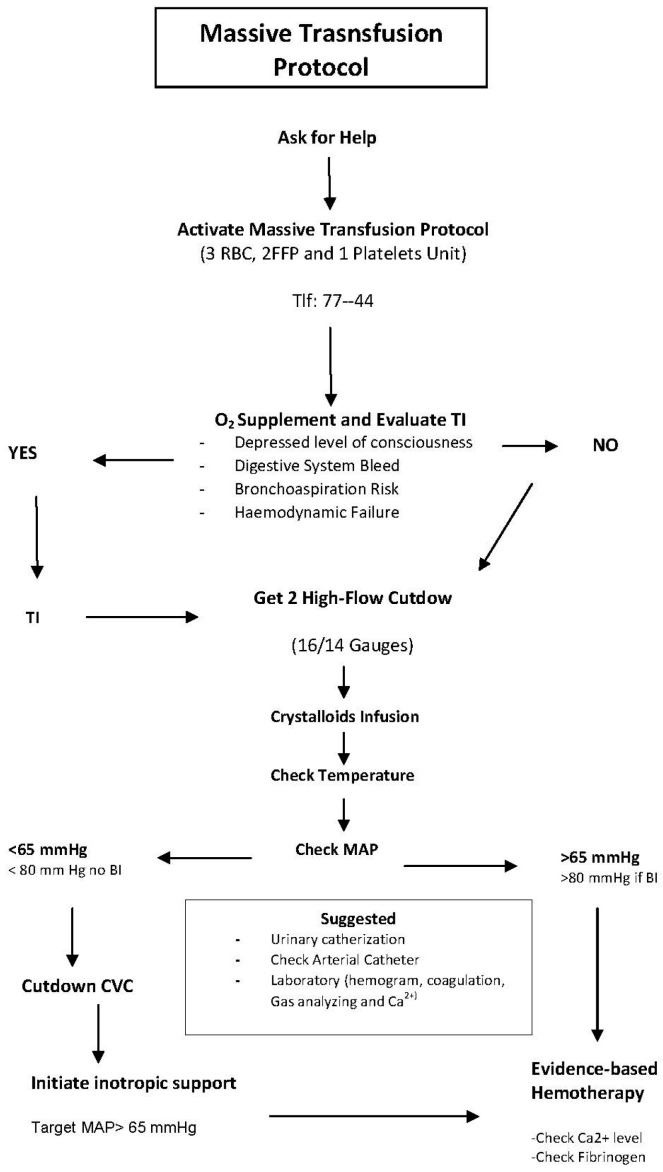
Cognitive aid. Massive transfusion protocol. FRBCs: Red Blood Cells. FFP: Fresh Frozen Plasma. TI: Tracheal Intubation. BI: Brain Injury. MAP: Mean Arterial Pressure. CVC: Central Venous Catheter.

Additionally, the need for a unified airway management protocol was identified to standardize criteria and streamline preparation. The massive transfusion protocol (see Figure 2) and the protocol for differentiating urgency/emergency in triage (see Table A1) were also revised. To further support process standardization, cognitive aids and visual tools were introduced, providing accessible resources for health professionals (see Table A2).

Among the tools implemented was the SBAR technique—a structured communication model designed to ensure accurate and efficient transfer of patient information. SBAR consists of four components:

Situation: Describe the current situation, including name, role, unit, and relevant changes in the patient’s condition or treatment plan.

Background: Provide clinical background such as age, sex, diagnosis, and ongoing treatments.

Assessment: Present an evaluation of the patient’s condition based on signs and symptoms.

Recommendation: Offer a recommendation or request specific instructions.

This tool has been successfully disseminated and standardized within the unit, improving communication between professionals and across care units such as the mobile ICU and intensive care unit.

The workshops also led to the formation of a multidisciplinary working group tasked with reviewing the triage process in the emergency department. This included evaluating the personnel involved and refining assessment criteria to optimize patient flow and prioritization.

Student evaluations were completed by the 112 participants and were very positive, with an average of 4.96 ± 0.268 out of 5 in relation to the objectives and contents of the course and an average of 4.87 ± 0.426 in relation to the methodology and documentation.

## 4. Discussion

We believe that effective crisis management requires specific skills that can be identified, taught, and practiced. There are well-established examples in fields such as aviation and the military, as well as in medical specialties like anesthesiology, where professionals operate under similar conditions of dynamism, pressure, complexity, uncertainty, and risk [1,5,7,13]. In this context, it is essential to actively address human factors and errors, and to develop strategies that can be applied across emergency services. In highly complex environments, experience and knowledge alone are often insufficient. Mistakes frequently occur in dynamic situations, many of which could be prevented through prior training [14,15,16].

Theoretical knowledge alone is not enough in crisis management; practical training is equally important [5,13]. Resource management during critical events requires structured analysis and reflection (debriefing), guided by experienced instructors following simulation-based scenarios. These sessions allow participants to evaluate their actions and reflect on cognitive processes, psychomotor skills, and emotional responses to enhance future performance. We firmly believe that this approach significantly enhances learning and facilitates the transfer of acquired skills to real clinical settings, or at least initiates actions to improve service delivery (e.g., protocols and cognitive aids). Importantly, this is achieved without compromising patient or staff safety [12,17,18,19,20,21,22].

The Advanced Simulation Centre of Integrated Care Management in Ciudad Real not only reviews clinical practices in response to critical incidents but also proactively identifies systemic risks to patient safety. Communicating these risks to relevant stakeholders (e.g., Risk Commission and hospital management) enables a proactive approach to safety and contributes to a safer healthcare organization [23].

The discussion phase of the debriefing process is crucial, involving deep, transparent, and shared reflection on individual and team behaviors. A psychologically safe environment is established to support this analysis. Instructors guide participants through effective debriefing techniques, such as “Debriefing with Good Judgment,” which begins with curiosity and seeks to understand the rationale behind specific actions. This method allows for tailored feedback based on the gap between expected and observed performance, helping participants develop more effective responses in future scenarios [24,25,26,27,28,29].

The implementation of brief, on-site training sessions incorporating interprofessional briefings could be an effective strategy for improving collaboration among healthcare professionals without significantly disrupting clinical workflows [30].

Simulation-based training also serves as a tool for identifying potential patient safety risks, aiming to maximize its impact on both patients and healthcare professionals. By incorporating the health risk management cycle into the simulation process, risks can be proactively identified and analyzed before harm occurs. This methodology supports the implementation of improvement strategies that can be extended across clinical and administrative processes within the organization. In addition, these workshops may serve as a valuable mechanism for identifying operational deficiencies within the department and for promoting the development of new clinical protocols or guidelines aimed at enhancing patient care and management [31]

This study presents several important limitations that should be acknowledged. First, the absence of a control group limits the ability to draw causal inferences regarding the effectiveness of the intervention. Second, the voluntary nature of participation may have introduced selection bias, as individuals more motivated or interested in simulation-based training may have been more likely to enroll. Third, the use of self-reported measures—particularly those related to satisfaction and perceived learning—may be subject to measurement bias. Fourth, no checklists or objective performance measures were collected during the simulation scenarios, which limits the ability to assess changes in technical or non-technical skills. Fifth, the findings are based on data from a single institution, which restricts the generalizability of the results to other healthcare settings. Sixth, it has not yet been possible to study the actual adoption of the protocols developed during the workshops, and no implementation metrics are currently available, as the process remains in a developmental phase. Finally, this study did not include patient-level outcome data, which limits the ability to assess the direct impact of the training on clinical practice and patient safety.

## 5. Conclusions

This study presents the implementation of innovative simulation-based training techniques for crisis resource management in the emergency department. The initiative showcases the use of simulation as an increasingly valuable educational tool that facilitates effective teamwork training. Through this project, healthcare professionals were able to develop competencies that are otherwise difficult to acquire in conventional training environments, ultimately enhancing workplace safety—particularly in areas such as the massive transfusion protocol.

The integration of this methodology into the continuous professional development of healthcare teams has introduced new stimuli and motivation, fostering a culture of safety and collaboration within emergency care settings.

## Figures and Tables

**Table 1:** Key points in resource management in crises during critical episodes in anesthesia and emergency medicine.

CRM Anesthesia	CRM in Emergency Medicine
✓ Know the environment. ✓ Anticipate and plan. ✓ Ask for help soon. ✓ Exercise leadership and know how ✓ to follow it. ✓ Distribute the workload. ✓ Mobilize all available resources. ✓ Communicate efficiently. ✓ Use all available information. ✓ Prevent and manage fixing errors. ✓ Double cross-checks. ✓ Use cognitive aids. ✓ Re-evaluate periodically. ✓ Good teamwork. ✓ To distribute the attention in a ✓ judicious way. ✓ Establish priorities dynamically.	✓ Situational awareness ✓ Anticipate, share and review plan. ✓ Provide effective leadership. ✓ Ensure clarity of roles and good teamwork. ✓ Communicate effectively. ✓ Ask for help early. ✓ Allocate attention wisely, avoid fixation. ✓ Distribute workload, monitor and support team members.

**Table 2:** Protocol for the design of simulated scenarios.

Planning of Teaching Objectives. (Both clinical and non-technical)
Brief summary for the staff just before starting the scenario with the key points of it.
Elements needed for scenario preparation.
Characteristics of the simulation room (placement of material, etc.). Type of simulator required and elements for its characterization. Necessary monitoring. Auxiliary material (tables, sera, medication, defibrillator, etc.). Medical history (computer with electronic medical history, graph of constants, analytics, imaging tests, etc.). Set up the stage as realistically as possible.
Brief narrative description of the scenario for all participants.
Staff involved in the stage: actors, confederates and participants. Summary of the key points of the scenario for the simulation personnel. Script of the scenario (Presentation of the clinical situation, development of the clinical case with the most relevant aspects, subsequent evolution until its resolution according to the interaction between the participants…). Parameters for the patient simulator (Constants that were modified according to the evolution of the case).
Guide for analysis and debate (emotions, orientation, key points to be dealt with, although they will ultimately depend on the concerns of the participants).
Closing the debate with a brief summary of the case and tools to take away.

**Table 3:** Satisfaction Survey Items Evaluated on a 0–5 Scale.

Item	Statement
1	The course’s alignment with the stated objectives
2	The course content met the participant’s expectations
3	Time distribution across topics was appropriate in relation to their importance and interest
4	The publicity, selection, and notification process was appropriate
5	Student support during the course was adequate (e.g., materials and inquiries)
6	Classroom facilities and technical resources were adequate (e.g., computers, projectors, web connections, and software)
7	The course schedule was appropriate
8	The course duration or time allowed for completion was appropriate in relation to its content
9	Participants felt their opinions were respected during the sessions
10	Simulation promoted self-confidence
11	Simulation was beneficial in linking theory to practice
12	Debriefing facilitated reflection on the cases
13	Simulation helped improve communication and teamwork skills
14	Simulation contributed to enhancing professional competence
15	Simulation increased self-assurance
16	Simulation was useful for improving routine professional practice
17	The learning environment encouraged participation and stimulating discussions
18	The instructor consistently provided constructive feedback after each simulation session
19	Participants were given tools to achieve better performance in the future

## Data Availability

The original contributions presented in this study are included in the article. Further inquiries can be directed to the corresponding author.

## Public Involvement Statement

All participants in the training workshops were healthcare professionals working in the Emergency Department of the General University Hospital of Ciudad Real (Healthcare Service of Castilla-La Mancha). Therefore, no members of the public (patients, consumers, or carers) were involved in any aspect of this research.

## Guidelines and Standards Statement

This study was conducted in accordance with institutional guidelines and ethical standards applicable to simulation-based educational research. All procedures followed the internal protocols of the Advanced Simulation Center of the General University Hospital of Ciudad Real and complied with the ethical principles outlined by the Healthcare Service of Castilla-La Mancha.

## Abbreviations

The following abbreviations are used in this manuscript:

CRM	Crisis Resource Management
ICU	Intensive Care Unit
SBAR	Situation–Background–Assessment–Recommendation
FRBC	Red Blood Cell
FFP	Fresh Frozen Plasma
TI	Tracheal Intubation
BI	Brain Injury
MAP	Mean Arterial Pressure
CVC	Central Venous Catheter

## Appendix A

**Table A1:** Protocol for triage in pediatric emergencies.

Urgency Level	Type of Urgency	Color Code	Maximum Waiting Time
1	Resuscitation	Red	Immediate
2	Emergency	Orange	10–15 min
3	Urgency	Yellow	30 min
4	Minor Urgency	Green	60–90 min
5	Non-Urgent	Blue	120 min

1. **Definition of Urgency and Emergency**

**Urgency:** A clinical situation that may lead to deterioration or pose a risk to the patient’s health or life depending on the time elapsed between its onset and the initiation of treatment.

**Emergency:** A critical situation with evident danger to the patient’s life requiring immediate intervention.

2. **Triage Definition**

Triage is a method used in emergency medicine for preliminary clinical assessment. It prioritizes patients before full diagnostic and therapeutic evaluation based on urgency/severity and available resources, aiming to maximize survival.

3. **Pediatric Triage System**

**A. Initial Clinical Assessment**

Use the Pediatric Assessment Triangle (PAT) for rapid evaluation.

**B. Identification of the Leading Symptom/Sign**

Focus on the symptom or sign that poses the greatest risk of deterioration during the waiting period.

**C. Priority Considerations Based on Reason for Consultation**

Avulsion of permanent tooth → Priority 2

Paraphimosis → Priority 2

Suspected intoxication → Priority 2–3

**D. Priority Modifiers**

Patients with the following characteristics should be considered Priority 3:

- Age under 4 months
- Underlying pathology (e.g., metabolic disease and diabetes)
- Risk of contagion (e.g., oncology patients)
- Disability (e.g., autism and chromosomal disorders)

**E. Vital Signs Monitoring**

Always include measurement of vital signs as part of the triage process.

**Table A2:** SBAR cognitive aid used in the simulation.

SBAR	Description	Example (Polytrauma Patient Requiring Transfusion)
Situation	What is happening right now? (Name, role, unit, relevant changes in patient’s condition)	“This is Dr. Lee from the Emergency Department. We have a polytrauma patient with ongoing hemorrhage and signs of shock.”
Background	What is the clinical background? (Age, sex, diagnosis, ongoing treatments)	“The patient is a 34-year-old male, involved in a motor vehicle accident. He has multiple fractures and abdominal trauma.”
Assessment	What do you think is the problem? (Signs, symptoms, clinical evaluation)	“He is hypotensive (BP 80/45 mmHg), tachycardic (HR 130 bpm), pale, and his hemoglobin is 7.2 g/dL. Bleeding is not controlled.”
Recommendation	What do you suggest or need? (Recommendation or request for instructions)	“I recommend activating the massive transfusion protocol immediately. Do you agree, or do you have any additional instructions?”

## Appendix B. Template for the Design of Simulation Scenarios

**SIMULATION SCENARIO TEMPLATE**

Emergency Simulation Workshop

**________________________________________**

**Scenario Name:** Sepsis

**Scenario Authors: [xxxx]**

**Date: November [xxxx]**

**Confidentiality:** Please keep the contents of this document confidential from participants to avoid interfering with the educational objectives.

**________________________________________**

**Learning Objectives**

1. **Clinical Objectives:**

a. Triage of a pediatric patient in the emergency department.

b. Identification of the clinical presentation.

c. Initial treatment measures.

d. Detection of changes in the initial clinical situation.

e. In case of progressive deterioration with loss of consciousness and pulse, recognize cardiac arrest and initiate basic life support (BLS).

f. Advanced life support (ALS) algorithm.

2. **Non-Technical Skills:**

- Leadership
- Prioritization of actions
- Communication and task distribution
- Resuscitation sequence
- Use of algorithms/cognitive aids (e.g., SBAR)

**________________________________________**

**Brief Scenario Summary**

A 4-month-old infant, previously admitted during the neonatal period for non-isoimmune jaundice (4 days), is brought by his mother to the emergency department due to a 24 h fever, which in the last 12 h has not responded well to antipyretics. The mother reports three episodes of vomiting at home and increasing lethargy and inactivity. No respiratory symptoms. On arrival, the infant is noted to be lethargic and pale, with delayed capillary refill.

**________________________________________**

**Personnel Involved in the Scenario**

**Simulation Room:**

- Lead Instructor (in control room)
- Associate Instructor (in control room or scenario room)
- Scenario Actors: the child’s parent

**Participants:**

- 1 Nursing assistant
- 2 Nurses
- Resident(s)
- 1/2 Attending physician

**________________________________________**

**Briefing Data**

Brief summary for staff just before starting the scenario, highlighting key points:

Winter night, midnight. A 4-month-old infant is brought to the emergency department by his mother due to fever (39 °C, 24 h), vomiting (3 episodes), and lethargy. The child is noted to be minimally responsive and markedly pale.

**Triage:** According to the Pediatric Assessment Triangle (PAT), the child is in decompensated shock (appearance and circulation), requiring immediate ABCDE assessment.

**________________________________________**

**Scenario Description for All Participants**

**Setting:** Pediatric Emergency Department. Weekend night shift. The team consists of two nurses and one nursing care technician. The resident physician is present in the ED, and the attending physician has just left for the meeting room.

A 4-month-old infant arrives with his mother due to fever, lethargy, and vomiting. Marked pallor is observed. During triage, decompensated shock is identified, and the patient is transferred to the assessment box.

**ABCDE assessment:**

- On undressing, petechiae and macules are observed on the chest, abdomen, and limbs. Sepsis is suspected.

**________________________________________**

**Scenario Preparation**

**(Room, devices, history, tests, and people)**

1. Pediatric emergency department. TRIAGE ROOM and subsequent ASSESSMENT BOX.
2. Monitors: pulse oximeter, monitor, blood pressure.
3. IV pumps: with saline solution for maintenance/basal needs.
4. Crash cart: in usual location.
5. Furniture: Standard ED equipment.
6. Simulator: Intubatable infant manikin.
7. Healthcare personnel and family involved: the child’s parent.
8. Other materials: appropriate resuscitation bag, triangular mask, oxygen therapy, endotracheal tube (4–4.5), laryngoscope, IV/intraosseous access, adrenaline, saline, bicarbonate, ventilator, ultrasound.
9. Relevant clinical history: previous admission for mild non-isoimmune jaundice.
10. Necessary tests: blood gas, chest X-ray.

**________________________________________**

**Scenario Phases Table**

**Phase**	**[Recommended Duration]**	**Patient**	**Confederates**	**Clinical History**	**Context**	**Key Actions**
**1. Framing**	[1 min]	4-month-old, lethargic, dressed and wrapped in a blanket for warmth	Parent brings the child, reports lethargy and poor response to antipyretics	Triage sheet	Arrival at ED, nurse at triage	Triage categorization
**2. Contextualization**	[1–3 min]	4-month-old, lethargic, irritable, intermittent crying, pale, delayed capillary refill	Parent expresses concern, prior neonatal admission, increased lethargy	Weight, age	Assess consciousness, monitor, gather history	1. Take the child 2.Assess consciousness 3. Monitor 4.Ask about history 5. Duration 6. Parent’s anxiety
**3. Presentation of Objectives**	[1–10 min]	Similarly to above	—	—	Resident plans care, examines patient	—
**4. Case Evolution**	[5–10 min]	Patient becomes more lethargic, unresponsive, vital signs deteriorate (SpO_2_ 80%, bradycardia 60 bpm)	Parent expresses anxiety: “He’s not moving, he’s cyanotic”	—	ABCDE, oxygen, IV access, fluids, antibiotics, BLS/ALS, call ICU	If delayed: cardiac arrest (asystole), parent distress
**5.Case Resolution**	[1–5 min]	If managed well: recovery, stabilization, transfer to ICU. If not: continued arrest, need for advanced resuscitation	ICU attending arrives, evaluates information transfer, organizes post-resuscitation care	—	—	—

**________________________________________**

**Key Points for Debriefing**

- **Emotions:** Anxiety, stress, nervousness, concern for the patient’s life
- **Exploration:**

**-Non-technical skills:** group dynamics in rare and unexpected situations, leadership, organization, task distribution, communication (including SBAR)

**-Technical skills:** pediatric triage, triage modifiers, BLS/ALS algorithms

- **Closure:**

-Review of performed or omitted techniques

-Review of the pediatric cardiac arrest algorithm

-Importance of communication, organization, and leadership

## Appendix C. Emergency Simulation Workshop Schedule

**Date**	**Time**	**Content**
03/11/XX	15:30–17:00	Introduction to clinical simulation
	17:00–17:30	Simulated Case: 4-month-old infant presenting with fever and lethargy. Sepsis. Cardiorespiratory arrest.
	17:30–18:30	Analysis and reflection on actions taken during the simulated case
	18:30–19:00	Simulated Case: 54-year-old woman with decreased consciousness, rescued from a pool. Drowning. Airway management.
	19:00–19:45	Analysis and reflection on actions taken during the simulated case
	19:45–20:15	Simulated Case: 35-year-old patient involved in a motorcycle accident. Respiratory difficulty, altered consciousness, pallor. Polytrauma. Massive transfusion.
	20:15–21:00	Analysis and reflection on actions taken during the simulated case
	21:00–21:30	Conclusions and closing session
